# Supplementary material for: Cvill6 and Cvill7: Potent and Selective Peptide Blockers of Kv1.2 Ion Channel Isolated from Mexican Scorpion Centruroides villegasi
Source: Toxins (Basel). 2025 Jun 4;17(6):279. doi: 10.3390/toxins17060279 (PMC12197678; doi:10.3390/toxins17060279)
Supplement: Supplementary file 1 [file toxins-17-00279-s001.zip › toxins-3611120-supplementary.pdf]

Supplementary Material

Cvill6 and Cvill7: Potent and Selective Peptide Blockers of Kv1.2 Ion Channel Isolated from Mexican Scorpion *Centruroides villegasi*

Kashmala Shakeel[1], Muhammad Umair Naseem[1], Timoteo Olamendi-Portugal[2], Fernando Z. Zamudio[2], Lourival Domingos Possani*[2], Gyorgy Panyi*[1]

[1]Department of Biophysics and Cell Biology, Faculty of Medicine, Research Center for Molecular Medicine, University of Debrecen, Egyetem ter. 1, Debrecen, 4032, Hungary
[2]Departamento de Medicina Molecular y Bioprocesos, Instituto de Biotecnologia, Universidad Nacional Autónoma de México, Av. Universidad 2001, Cuernavaca 62210, Mexico
*Corresponding authors

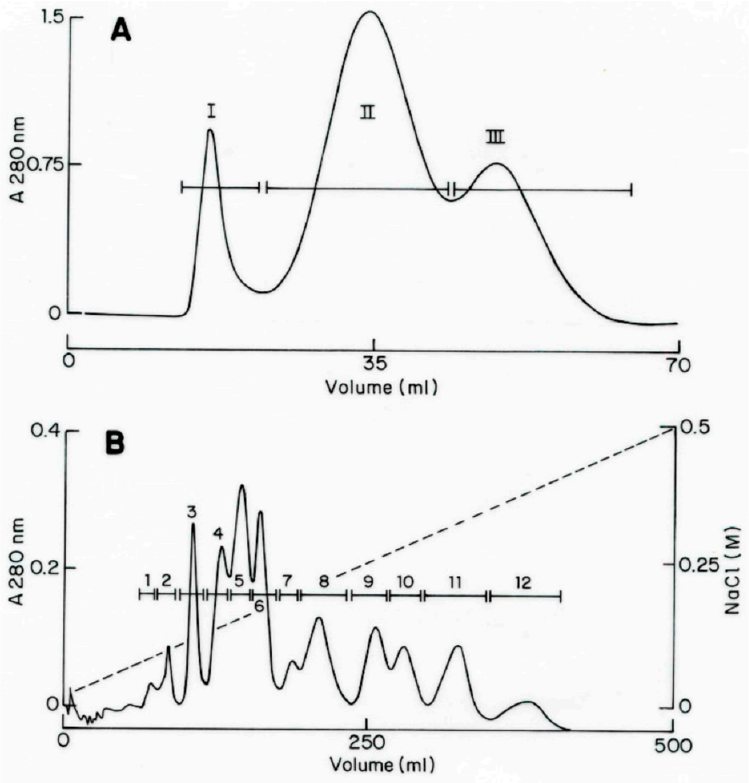

Figure S1. Chromatographic separation of the crude venom. (A) Separation of soluble venom of *C. villagasi* using Sephadex G-50. **(B)** Separation of F-II fraction from A through carboxymethylcellulose (CMC) column using a gradient of NaCl from 0-0.5 M for 1000 min. Adopted from Riaño-Umbarila et al. (2024) [40].

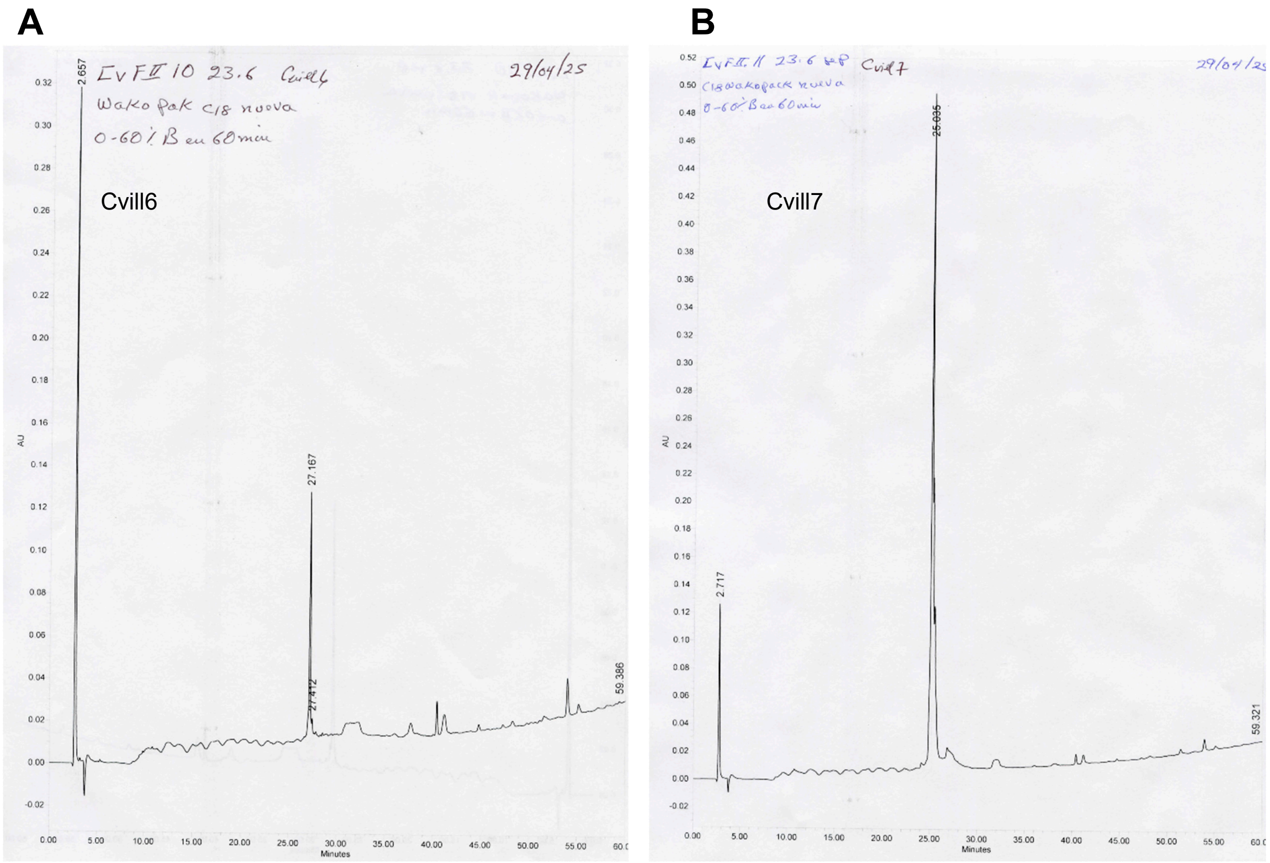

Figure S2. HPLC analysis of Cvill6 and Cvill7. **(A & B)** chromatograms of HPLC analysis using C_{18} column of Cvill6 (**A**) and Cvill7 **(B)** native purified peptides. Peptides were eluted with a 0-60% gradient of solution B (acetonitrile containing 0.12% TFA) in solution A (deionized water containing 0.12% TFA) over 60 min.

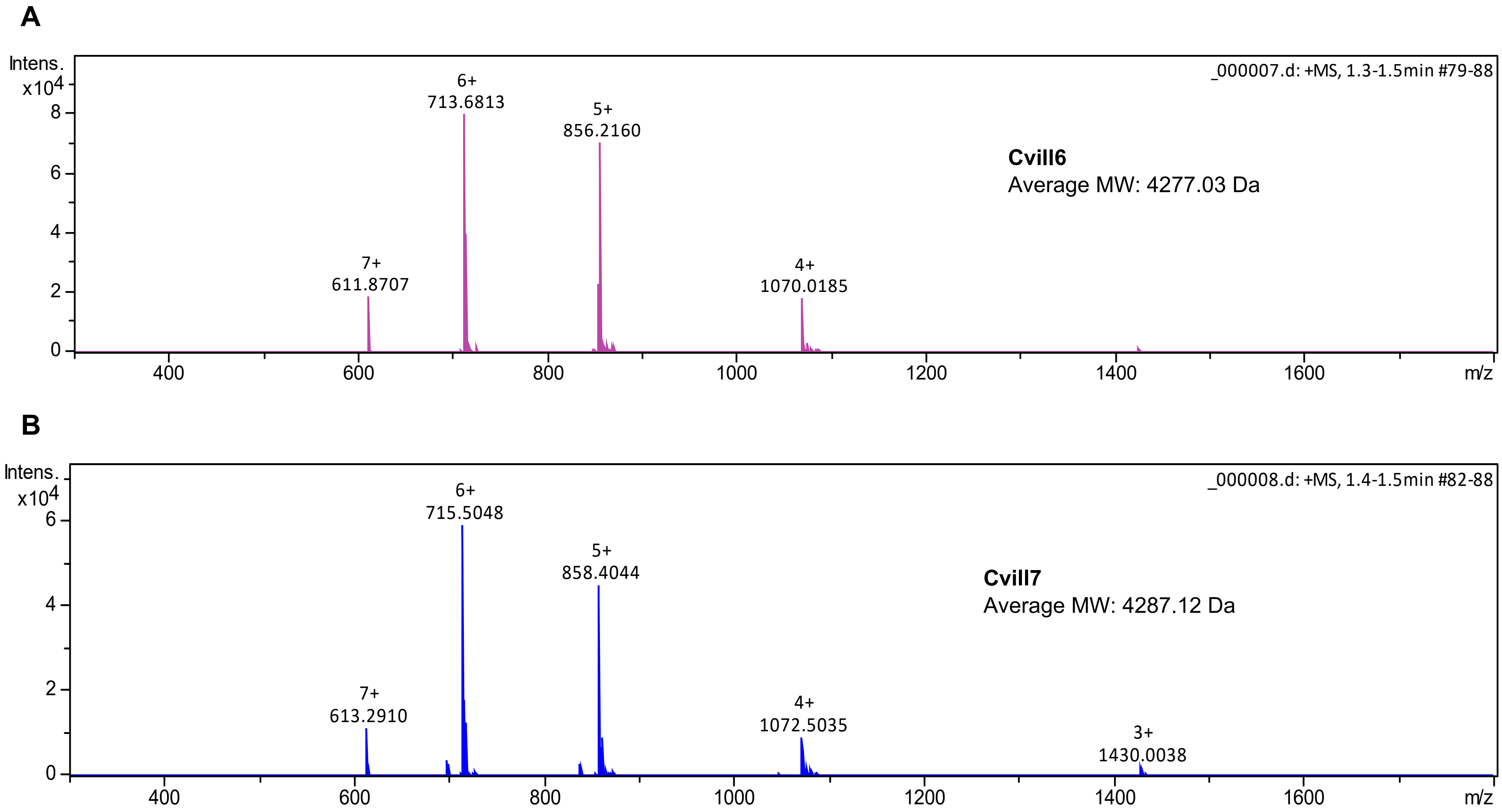

Figure S3. Mass spectrometry analysis of peptides Cvill6 and Cvill7. (A & B) Mass spectrometry analysis of Cvill6 **(A)** and Cvill7 **(B)** peptides using ESI QTOF-MS instrument (maXis II UHR ESI-QTOF MS, Bruker, Bremen, Germany). Average MW of peptides are given in inset.

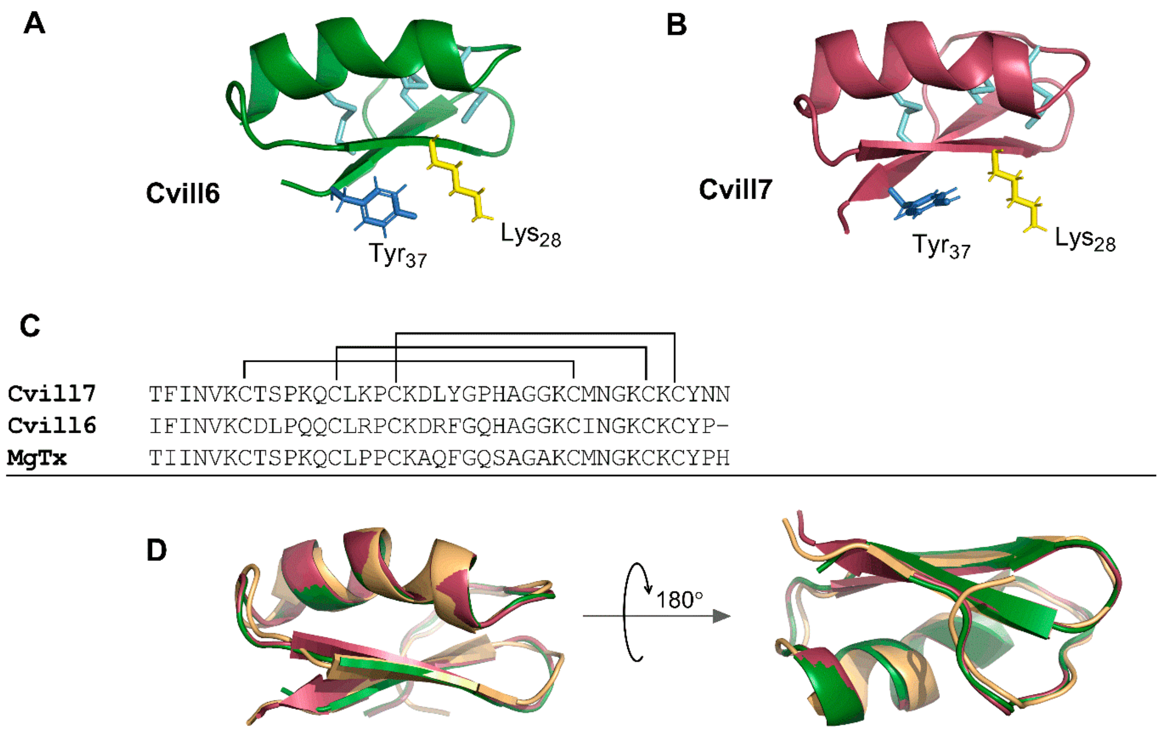

Figure S4. Sequence and structural alignments of Cvill6 and Cvill7. Predicted 3-D models of Cvill6 (A) and Cvill7 (B) obtained using Alphafold3. Ribbon representation (Cvill6 in forest green and Cvill7 in raspberry color) of backbone structure with dyad related residues indicated in colored (Tyr in blue and Lys in yellow) sticks and disulfide bonds shown in cyan sticks. (C) Primary sequence comparison of Cvill peptides and MgTx. Cysteines are shown in bold and similarity-based predicted disulfide connectivity are indicated above the sequence. (D) Structural alignment between predicted models of Cvill6 (shown in forest green), Cvill7 (shown raspberry color) and MgTx (PDB: 1MTX, shown in light orange color).

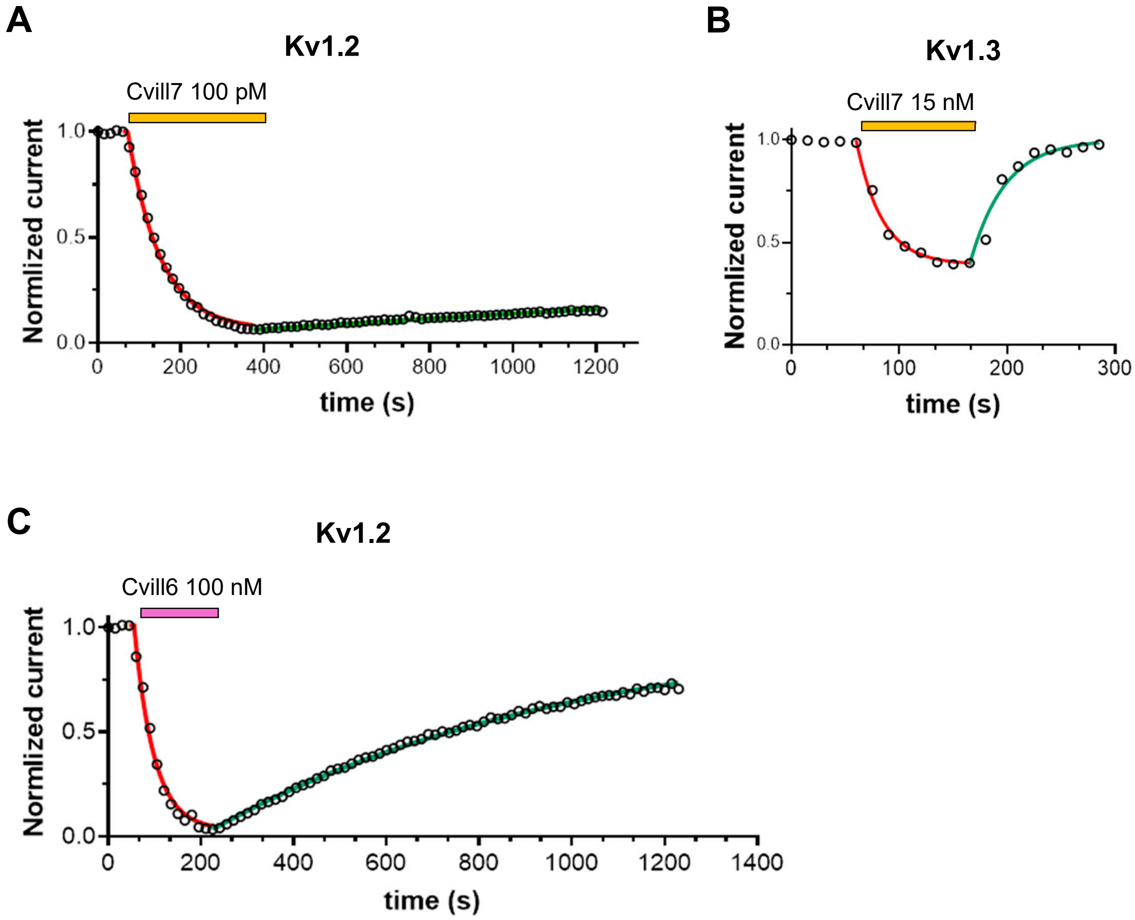

Figure S5. Analysis of block kinetics. Representative fits to determine the time constants of development and recovery of Kv1.2 **(A & C)** and Kv1.3 **(C)** current inhibition by Cvill7 (indicated with orange horizontal bars, **A-B**) and Cill6 (purple horizontal bar, **C**). The normalized peak currents (see the main text for detail) during the toxin application (wash-in procedure) were fitted using a single exponential decay function (the fitted lines are shown in red), yielding wash-in time constants (τ_{on}), Kv1.2: 75.3 s **(A)** and 38.7 s **(C)** for Cvill7 and Cvill6, respectively, and Kv1.3: 22.6 s **(B)** for Cvill7. The data points after application of toxin-free solution (wash-out procedure) were fitted single-exponential rise function (the fitted lines are shown in green) to give gave wash-out time constants (τ_{off}) for Kv1.2: 7380 s **(A)** and 776.4 s **(C)** for Cvill7 and Cvill6, respectively, and Kv1.3: 34.5 s **(B)** for Cvill7.
